# Longitudinal increase in total IgE levels in patients with adult asthma: an association with poor asthma control

**DOI:** 10.1186/s12931-014-0144-8

**Published:** 2014-11-20

**Authors:** Akihiko Tanaka, Megumi Jinno, Kuniaki Hirai, Yoshito Miyata, Hiroko Mizuma, Munehiro Yamaguchi, Shin Ohta, Yoshio Watanabe, Mayumi Yamamoto, Shintaro Suzuki, Takuya Yokoe, Mitsuru Adachi, Hironori Sagara

**Affiliations:** Department of Internal Medicine, Division of Allergy and Respiratory Medicine, Showa University, School of Medicine, 1-5-8 Hatanodai, Shinagawa-ku, Tokyo, 142-8666 Japan; Department of Allergy, Sanno Hospital, Clinical Research Centers for Medicine, International University of Health and Welfare, Tokyo, Japan

**Keywords:** IgE, Longitudinal change, Severe asthma, Aspergillus, house dust mite

## Abstract

**Background:**

Immunoglobulin (Ig) E is well-known to play a critical role in allergic diseases. We investigated the association between longitudinal change in total IgE level and the asthma control in patients with adult asthma.

**Methods:**

For this retrospective study, 154 patients with asthma aged 21–82 years were recruited from the allergy and pulmonary units of the Showa University Hospital. Data on longitudinal changes in IgE over the preceding 10 years were collected and logarithmically transformed. Associations between longitudinal change in IgE and clinical characteristics including asthma control test (ACT) score, asthma control, pulmonary function test, and antigen specific IgE, were assessed.

**Results:**

Patients with increased IgE tended to have significantly higher mean age, more episodes of acute exacerbation within a year, lower ACT scores, and used oral corticosteroids more frequently than those with decreased or unchanged IgE. The prevalence of uncontrolled asthma was higher in patients with increased IgE than in those with decreased or unchanged IgE. Mean %FEV_1_ and FEV_1%_ were lower in patients with increased IgE than in those with decreased or unchanged IgE. Moreover, the prevalence of *Aspergillus*-specific IgE was higher in patients with increased IgE than in those with decreased or unchanged IgE.

**Conclusions:**

These data suggest that a longitudinal increase in total IgE is associated with both poor asthma control and *Aspergillus*-specific IgE in patients with adult asthma.

## Introduction

Immnoglobulin E (IgE), the fifth and last immunoglobulin to be identified, was first reported in 1966 [1]. Measurements of total and antigen-specific IgE are helpful for diagnosing allergic diseases. IgE is a critical factor for the development of bronchial hyperresponsiveness in asthmatics [2]. Epidemiological studies have shown that total IgE level is higher in patients with asthma, particularly in children, than in non-asthmatics [3-5]. Recently, a longitudinal analysis of European Community Respiratory Health Survey data showed that total IgE was associated with new-onset asthma among atopics [6]. Meanwhile, the association between total IgE level and non-atopic asthma remains controversial [7,8].

Total IgE level is higher in children with severe asthma than in those with mild to moderate asthma [9]. In The Epidemiology and Natural History of Asthma: Outcomes and Treatment Regimens study, an association between a high level of total IgE and asthma severity was observed among younger subjects with difficult-to-treat or severe asthma [10,11]. In a study conducted by de Marco et al., a high level of IgE was a strong predictor of moderate-to-severe asthma among 856 European adult patients with asthma [12]. However, recent global multicenter clinical trials, such as the Severe Asthma Research Program [13] and the European Network For Understanding Mechanisms of Severe Asthma [14], showed no correlation between asthma severity and levels of total and antigen-specific IgE, raising suspicion that IgE may not play a role in the deterioration of asthma control in patients with adult asthma. In recent years, a recombinant humanized monoclonal anti-IgE antibody, omalizumab, has been used in patients with allergic severe asthma [15,16]. Its effectiveness reminds us of the functional importance of IgE in severe asthma.

In general, serum total IgE concentration seems to peak in early adolescence and decrease with age [17-19]. However, longitudinal change in IgE is heterogeneous among patients with adult asthma. Although there have been several large-scale cross-sectional studies of total IgE and antigen-specific IgE [20,21], only one multicenter study has assessed longitudinal change in total IgE and prevalence of IgE sensitization in the same population [22]. This study showed an overall decrease in total IgE over 10 years, in line with previous cross-sectional studies [17-19]. Meanwhile, few studies have interpreted longitudinal change in total IgE in individuals.

The aim of the present study was to investigate the association between longitudinal change in total IgE and asthma control to elucidate the role of longitudinal change in IgE in patients with asthma.

## Methods

### Study design

This was a retrospective study conducted in the allergy and pulmonary units of the Showa University Hospital, Tokyo, Japan, between January 2011 and December 2012. The study protocol was approved by the Showa University ethics committee and written informed consent was obtained.

### Study subjects

Out of 854 asthmatic patients, we recruited 154 patients, whose longitudinal changes in total IgE in the preceding 10 (±2) years could be obtained. All patients had been regularly followed at Showa University Hospital. Individuals with chronic obstructive pulmonary disease (COPD) or other lung disease, smoking history greater than 20 pack-years, being treated with omalizumab, vocal cord dysfunction, or neurological disease were excluded. No participants, even those who had high IgE level, had parasitic infections. Asthma was diagnosed based on cough, breathlessness or dyspnea, and demonstration of airflow variability. The latter was determined according to a reversible airflow limitation that represented an increase in forced expiratory volume in 1 s (FEV_1_) of 12% or 200 mL after the inhalation of salbutamol.

### Data collection

Baseline data including medical history, Asthma Control Test (ACT) score, spirometry, fractional exhaled nitric oxide (FeNO), percent peripheral eosinophils, total and specific IgE [*Dermatophagoides pteronissinus* (house dust mite: HDM), *Cryptomeria japonica* (cedar), *Ambrosia artemisiifolia* (ragweed), *Candida albicans* (candida), *Aspergillus fumigatus* (aspergillus), *Alternaria alternata* (alternaria), dog dander, cat dander, *Bombyx mori* (moth), and *Blatta orientalis* (cockroach)] were collected at enrollment. The definition of atopic type was being positive to crude house dust-specific IgE.

Asthma control was assessed using the validated Japanese version of the ACT. Patients were subjectively evaluated for the degree of impairment caused by their asthma during the preceding 4 weeks by responding to five questions using a five-point scale.

Spirometry was performed using an AS-302 spirometer (Minato Medical Science Co., Ltd., Osaka, Japan) in accordance with American Thoracic Society/European Respiratory Society guidelines [23,24] to determine FEV_1_, forced vital capacity (FVC), and FEV_1_/FVC (FEV_1%_). The highest value from three technically satisfactory attempts was recorded. FEV_1_ and FVC values were expressed as a percentage of the predicted value.

FeNO was measured by a portable device (NIOX MINO, Aerocrine AB, Solna, Sweden) at an expiratory flow rate of 50 mL/s for 10 s.

Serum total IgE levels, measured with a fluorescent enzyme immunoassay (ImmunoCAP-FEIA, Phadia, Freiburg, Germany) were transformed logarithmically to normalize their distribution. A zero was replaced with a value half of the lowest value observed before log transformation. Antigen-specific IgE was also measured with ImmunoCAP-FEIA, and levels higher than class 2 were considered as positive. The longitudinal change in total IgE (ΔIgE) was calculated as log total IgE at present – log total IgE 10 years ago. An increase or decrease in total IgE was arbitrarily designated as a variation of >0.15 log10 kU/L.

### Statistical analyses

Results were expressed as mean ± SD for continuous variables. FeNO values were transformed logarithmically to normalize their distribution. All analyses were performed using JMP version 10 (SAS Institute Inc., Cary, NC, USA). Differences in the continuous variables between groups were tested by analysis of variance (ANOVA); if the differences between groups were significant, comparisons were made by unpaired *t*-test. Categorical variables were analyzed with *χ*^2^ tests. A value of *P* <0.05 was considered significant for all statistical assessments.

## Results

### Background

One hundred and fifty-four patients with asthma with a median age of 62.5 years (range 21–82) were included. The mean BMI was 23.1 ± 3.6, and 62 patients (40.3%) were men. Thirteen patients (8.4%) were current smokers, and 31 patients (20.1%) were ex-smokers at the time of enrollment. The mean ACT score and FeNO were 20.7 ± 3.7 and 51.3 ± 42.9 ppb, respectively. In the pulmonary function test, the mean FVC and FEV_1_ were 2.5 ± 0.9 L (%FVC: 88.2 ± 19.3%), and 1.8 ± 0.8 L (%FEV_1_: 75.0 ± 23.0%), respectively.

**Figure 1:**
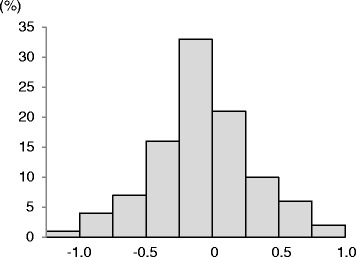
**Distribution of the longitudinal change in IgE across 0.25 in patients with adult asthma.**

Mean total IgE was 2.43 ± 0.65 log_10_ kU/L 10 years ago and 2.37 ± 0.66 log_10_ kU/L at the time of the study. An increase or decrease in IgE was arbitrarily designated as a variation of >0.15 log_10_kU/L. Figure 1 displays the distribution of patients according to longitudinal change in IgE.

Demographic details for the patient population are shown in Table 1. No significant differences were observed in gender, BMI, percent adult onset, smoking status, atopic type, pet ownership, allergic rhinitis, pollinosis, eosinophils, or FeNO between the groups. However, there were significant differences in age, prevalence of patients treated at levels higher than Step 4, frequency of use of on-demand oral corticosteroid (OCS), episode of acute exacerbation within a year, and pre-IgE level between the groups. Mean age was significantly higher for patients with increased IgE than for those with decreased or unchanged IgE. However, in this study, there was no significant correlation between ΔIgE and age (data not shown). Mean pre-IgE level was significantly lower in patients with increased IgE than in patients with decreased IgE.

**Table 1:** **Subject demographics and patient characteristics**

	**Decrease (N = 63)**	**No change (N = 50)**	**Increase (N = 41)**	**p value**
Age (years)	61.3 ± 14.6*	59.5 ± 13.7**	67.8 ± 11.2	0.011†
Gender (M/F)	27/36	21/29	14/27	NS
BMI	23.7 ± 0.4	23.2 ± 0.5	22.1 ± 0.5	NS
Adult onset, n (%)	45 (71.4)	39 (78.0)	34 (82.9)	NS
Smoking status, never/ex/current (%)	46/12/5 (73.0/19.1/7.9)	38/8/4/ (76.0/16.0/8.0)	26/11/4/ (63.4/26.8/9.8)	NS
Atopic type, n (%)	42 (66.6)	30 (60.0)	21 (51.2)	NS
Pet owner, n (%)	16 (25.4)	11(22.0)	9 (21.9)	NS
Allergic rhinitis, n (%)	30 (47.6)	23 (46.0)	15 (36.5)	NS
Pollinosis, n (%)	27 (42.8)	24 (48.0)	21 (51.2)	NS
Treatment with higher than Step 4, (%)¶	17 (26.9)	14 (28.0)	20 (48.78)	0.045‡
OCS on demand, none/rare/frequent (%) ‖	35/22/6 (55.6/34.9/9.5)	40/8/2 (80.0/16.0/4.0)	23/12/6 (56.1/29.3/14.6)	0.046‡
Episode of acute exacerbation within a year	15 (23.8)	7 (14.0)	15 (36.6)	0.042‡
Eosinophils, % of WBC	5.45 ± 0.48	4.75 ± 0.54	5.49 ± 0.60	NS
Pre-IgE, IU/L	1020.6 ± 1654.8	873.5 ± 2018.3	695.8 ± 2555.9	-
Pre-IgE (log-transforn), IU/L	2.59 ± 0.08***	2.40 ± 0.09	2.22 ± 0.10	0.014†
FeNO, ppb	52.2 ± 5.6	50.7 ± 6.3	50.5 ± 7.0	NS

Values are Mean ± SDs or medians (%).

†p values given for the ANOVA.

‡p values given for the Pearson *χ* ^2^ test.

*p < 0.05 or **p < 0.01 or ***p < 0.001 versus increase.

¶ Treatment step is based on the Global Initiative for Asthma (GINA) guideline.

‖ none: never, rare: less than once a month, frequent: once or more a month.

OCS: oral corticosteroids.

### ACT

**Figure 2:**
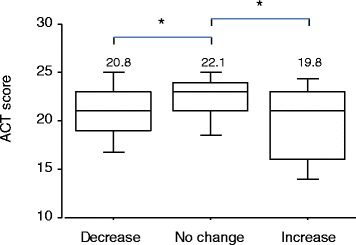
**ACT scores of patients with asthma.** Mean ACT scores are shown on the top of the bars. ACT comprises five questions to assess activity limitation, shortness of breath, nighttime symptoms, use of rescue medication, and patient’s overall rating of asthma control over the previous 4 weeks. *: Significant difference (P <0.01) versus the unchanged IgE group, as determined by *t*-test following a one-way analysis of variance (ANOVA).

To identify patients with poor asthma control, several tools can be used globally, one of which is ACT. This test comprises five questions to assess activity limitation, shortness of breath, nighttime symptoms, use of rescue medication, and the patient’s overall rating of asthma control over the previous 4 weeks. Mean ACT score was significantly lower for patients with increased IgE (Figure 2, P =0.006) and for patients with decreased IgE (Figure 2, P =0.007) than for patients with no change in IgE.

### Control level

**Figure 3:**
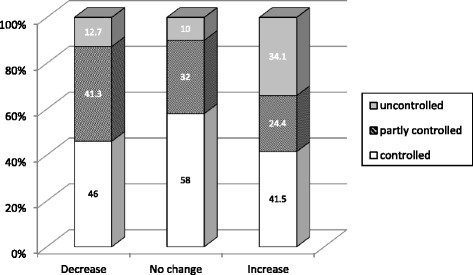
**Distribution of patients according to asthma control level.** Asthma was identified as controlled, partly controlled or uncontrolled based on the categorical scale of Global Initiative for Asthma. Low data were shown in the bars. A *χ* ^2^ test demonstrated a significant difference between the groups (P =0.014).

The Global Initiative for Asthma (GINA) guidelines, the most widely used asthma guidelines in the world, emphasize the importance of evaluating asthma control rather than asthma severity. The GINA guidelines provide a categorical scale for assessing asthma control, consisting of well-controlled, partly controlled, or uncontrolled asthma [25]. Asthma control levels of the patients in this study are shown in Figure 3. Significant differences were observed between the groups (P =0.014). In particular, 34.1% of the patients with increased IgE had uncontrolled asthma, compared with 12.7% and 10.0% of patients with decreased and unchanged IgE, respectively.

### Pulmonary function test

**Figure 4:**
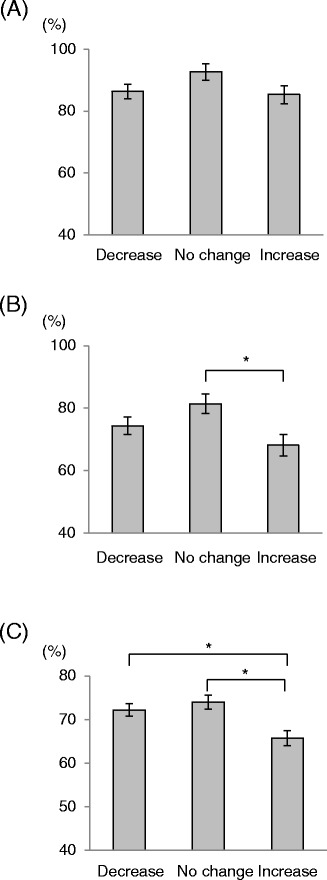
**Pulmonary function tests in patients with asthma.** %FVC **(A)**, %FEV_1_ **(B)**, and FEV_1%_ **(C)** are shown. The highest value from three technically satisfactory attempts was recorded. *: Significant difference (P <0.01) versus the indicated group, as determined by *t*-test following a one-way ANOVA.

Lung function, as an assessment of variable airway obstruction, is an objective of asthma control. There was no significant difference in %FVC between the groups (Figure 4A). In contrast, significant differences were observed in %FEV_1_ and FEV_1%_. The patients with increased IgE had lower %FEV_1_ than did the patients with no change in IgE (Figure 4B, P =0.006), and lower FEV_1%_ than patients with decreased or unchanged IgE (Figure 4C, P <0.001).

### IgE specific to inhaled antigens

The prevalence of IgE specific to inhaled antigens is shown in Table 2. There was no significant difference between the three groups for HDM, cedar, ragweed, candida, alternaria, dog dander, cat dander, moth, and cockroach. In contrast, a significant difference was found for aspergillus (P <0.001). The patients with increased IgE had a higher prevalence of aspergillus sensitization than other groups. In addition, patients who were positive to aspergillus-specific IgE had higher ΔIgE than patients who were negative for aspergillus-specific IgE (0.123 ± 0.36 vs −0.098 ± 0.41, P =0.008).

**Table 2:** **Numbers and percentages of patients positive for antigen-specific IgE**

	**HDM**	**cedar**	**ragwood**	**candida**	Aspergillus*	**alter-naria**	**dog dander**	**cat dander**	**moth**	**cock-roach**
decrease (N = 63)	42/63 (66.6%)	29/63 (46.0%)	5/63 (7.9%)	11/63 (17.4%)	4/63 (6.3%)	3/63 (4.7%)	11/47 (23.4%)	11/47 (23.4%)	13/50 (26.0%)	6/47 (12.7%)
no change (N = 50)	31/50 (62.0%)	32/50 (64.0%)	1/50 (2.0%)	8/50 (16.0%)	5/50 (10.0%)	1/50 (2.0%)	7/33 (21.2%)	8/33 (24.2%)	10/34 (29.4%)	4/22 (18.1%)
increase (N = 41)	22/41 (53.6%)	24/41 (58.5%)	6/41 (14.6%)	10/41 (24.3%)	15/41 (36.5)	5/41 (12.1%)	5/32 (15.6%)	5/32 (15.6%)	7/31 (22.5%)	6/29 (31.0%)

The denominator is the number of tested patients and the numerator is the number of patients who had a positive result. *: A *χ* ^2^ test demonstrated a significant difference between the groups (P <0.001).

## Discussion

In the present study, we found that patients with a longitudinal increase in total IgE used on-demand OCS more frequently, were more likely to have been treated at levels above Step 4, had more episode of acute exacerbation within a year, had lower ACT scores, and were more likely to have uncontrolled asthma than patients with a longitudinal decrease in IgE or no change in IgE. In addition, more airway obstructions were observed among patients with a longitudinal increase in IgE. These results suggest that a longitudinal increase in IgE is associated with poor asthma control. To our knowledge, this is the first study to investigate the clinical role of longitudinal change in total IgE in patients with adult asthma and to demonstrate the possible association between a longitudinal increase in IgE and poor control of adult asthma. Recent global trials showed no association between absolute IgE levels and asthma severity in patients with adult asthma [13,14]. Considering these previous global trials, our interpretation of the present study is that a measurement of longitudinal change but not absolute value in total IgE predicts, to some extent, asthma control in clinical practice.

Longitudinal changes in IgE vary considerably among individual in patients with adult asthma, as shown in Figure 1. We stratified adult asthma patients according to the longitudinal change in IgE to identify characteristics of patients with increased IgE. We found that the mean age of patients with a longitudinal increase in IgE was higher than that of patients with a decrease or no change in IgE, suggesting that an increase in total IgE is associated with aging among patients with uncontrolled asthma. Previous epidemiological studies showed that mean total IgE levels in patients with asthma decreased with age [20,26]. The participants in these studies were younger, (mostly less than 60) than those who enrolled in our study. In addition, these studies were cross-sectional rather than longitudinal. Thus, the results of these studies cannot be easily compared with our data. Further investigation of longitudinal changes in total and antigen-specific IgE in adult asthma, particularly over the age of 60, is needed.

Previous studies have shown that males and current smokers have higher IgE levels than females and non-current smokers, respectively [27-29]. In the current study, there were no significant differences in gender or current smoking status between three groups (Table 1). The reasons for this are still unclear, but one possible reason for the lack of a difference in smoking status is that participants whose smoking history was greater than 20 pack-years were excluded for exclusion of COPD.

The total IgE level reflects both specific and unspecific IgE. Matricardi et al. followed total and allergen-specific IgE levels in children from birth to 13 years of age [30]. This study revealed that the evolution of total IgE was extremely heterogeneous but was parallel with that of allergen-specific IgE from the age of 5 onwards. Furthermore, Carsin et al. demonstrated that total IgE level is related to new-onset asthma, but this association is almost entirely explained by specific IgE [31]. In the present study, specific IgE to HDM was the most prevalent, followed by cedar-specific IgE. The prevalence of specific IgE to HDM and cedar did not differ between groups, indicating that these antigen-specific IgE did not contribute to the longitudinal increase in total IgE. The prevalence of specific IgE to fungi, such as aspergillus and alternaria, was higher in the increased IgE group than in the other two other groups. In particular, significant difference was observed for aspergillus-specific IgE. This suggests that specific IgE to fungi, especially aspergillus, might have contributed to the longitudinal increase in total IgE and also to poor control in the increased IgE group. This is clinically plausible, since sensitization to aspergillus is one of the factors that results in difficult-to-treat asthma, such as allergic bronchopulmonary aspergillosis (ABPA) and severe asthma with fungal sensitization (SAFS) [32]. In addition, a previous study showed that specific IgE to *Aspergillus fumigatus* contributed to an increased risk of adult onset of asthma [33]. To the best of our knowledge, this is the first report indicating the association between aspergillus-specific IgE and a longitudinal increase in total IgE in patients with adult asthma.

There are several limitations to our study. First, although we showed that patients with increased IgE had a higher prevalence of aspergillus-specific IgE than patients with decreased or unchanged IgE, a longitudinal change in aspergillus-specific IgE could not be shown. Thus, to demonstrate directly the contribution of aspergillus-specific IgE to the increase in total IgE and poor control of asthma, we should assesse the association between longitudinal change in aspergillus-specific IgE and longitudinal change in total IgE and control levels.

Second, the treatments of patients were not taken into consideration in this study. The treatments of all patients enrolled in this study have changed, usually a number of times, during the past 10 years. Therefore, we could not account for the treatments in our analysis. It remains unclear how treatments affect longitudinal changes in total IgE and antigen-specific IgE.

In conclusion, this is the first study to investigate the clinical roles of longitudinal change in serum total IgE in patients with asthma. We demonstrated that longitudinal increase in total IgE is associated with poor asthma control and a higher prevalence of aspergillus-specific IgE in patients with adult asthma, suggesting a significant contribution of IgE in the pathophysiology of severe asthma. Further prospective analysis is needed to validate our data.

## Abbreviations

ACT: Asthma control test
ANOVA: Analysis of variance
BMI: Body mass index
Der p: Dermatophagoides pteronyssius
ECRHS: European community respiratory health survey
ENFUMOSA: European network for understanding mechanisms of severe asthma
FEIA: Fluorescent enzyme immunoassay
FeNO: Fractional exhaled nitric oxide
FEV_1_: Forced expiratory volume in 1 s
FVC: Forced vital capacity
GINA: Global initiative for asthma
Ig: Immunoglobulin
SAFS: Severe asthma with fungal sensitization
SARP: Severe asthma research program
TENOR: The epidemiology and natural history of asthma: outcomes and treatment regimens
